# Characteristics and outcome of rhabdomyolysis in acute ischemic stroke patients: a 10-year retrospective study

**DOI:** 10.7717/peerj.20645

**Published:** 2026-01-20

**Authors:** Zhen Wang, Bo Wang, Lirui Wang

**Affiliations:** 1 Shanghai Baoshan District Wusong Central Hospital, Shanghai, China; 2 Wusong Branch, Zhongshan Hospital Affiliated to Fudan University, Shanghai, China; 3 Tongji Hospital Affiliated to Tongji University, Shanghai, China

**Keywords:** Rhabdomyolysis, Acute ischemic stroke patients, Risk factors

## Abstract

**Introduction:**

While acute ischemic stroke (AIS) and rhabdomyolysis (RML) have been reported to co-occur, their clinical relationship and associated outcomes remain poorly understood. This study aimed to investigate the clinical characteristics and outcomes of patients with concurrent AIS and RML.

**Methods:**

A retrospective analysis was conducted on patients admitted to the Department of Neurology from January 2014 to December 2023. The clinical and laboratory indicators, as well as the prognosis at discharge, were assessed.

**Results:**

Among 9,360 AIS patients, 146 with RML (CK > 1,000 U/L) were assigned to the RML group, while 146 without RML formed the control group. Patients with RML had a higher incidence of comorbidities and acute complications, including diabetes (43.15% *vs.* 26.03%, *p* = 0.002), coronary heart disease (22.60% *vs.* 10.96%, *p* = 0.008), and acute kidney injury (35.36% *vs.* 5.48%, *p* < 0.001). Poor outcome (death or discharge against medical advice, DAMA) was significantly higher in the RML group than in controls (28.77% *vs.* 3.42%, *p* < 0.001). Multivariable logistic regression identified NIHSS score > 15 (OR = 4.932, 95% CI [1.902–12.794], *p* = 0.001), infection (OR = 5.897, 95% CI [1.550–30.112], *p* = 0.033), and elevated troponin I (>0.03 ng/ml; OR = 3.384, 95% CI [1.185–9.664], *p* = 0.023) as independent predictors of poor outcomes. However, RML itself was not an independent predictor.

**Conclusions:**

AIS patients with RML exhibited an increased poor outcome rate. While multivariable analysis identified NIHSS score >15, infection, and elevated troponin I as independent predictors, RML was not an independent risk factor. Given the observational design and the co-occurrence with severity markers, these associations should not be interpreted as independent effects of RML. These findings pertain to in-hospital outcomes only. Post-discharge functional endpoints (*e.g.*, 90-day modified Rankin Scale) were not available.

## Introduction

Acute ischemic stroke (AIS) remains a leading cause of mortality and long-term disability worldwide, despite significant advancements in diagnostic and therapeutic strategies over the past decade (Phipps & Cronin, 2020). In the United States, the incidence of AIS has decreased, yet it continues to impose a substantial economic burden globally (Phipps & Cronin, 2020; Herpich & Rincon, 2020). Rhabdomyolysis (RML), characterized by the rapid breakdown of skeletal muscle tissue and the subsequent release of intracellular components into the bloodstream, is a condition that can complicate the clinical course of AIS (Hsu, Chiu & Liao, 2009). The primary clinical manifestations of RML include muscle weakness, pain, and dark urine, with elevated creatine kinase (CK) levels serving as a critical diagnostic marker (Stahl, Rastelli & Schoser, 2020).

The co-occurrence of AIS and RML is increasingly recognized, notably in the setting of drug overdose (Hsu, Chiu & Liao, 2009; Kim et al., 2023). Although case reports describe RML in AIS, to our knowledge there are no clinical studies or systematic reviews on this topic to date. Several pathophysiological pathways may link these conditions. Prolonged immobilization after AIS, especially when muscles are maintained in shortened positions, can initiate a cascade that culminates in RML (Jalal, Gracies & Zidi, 2020). Second, statin therapy, widely used for secondary prevention after ischemic stroke, is a well-recognized, albeit rare, precipitant of RML (Montastruc, 2023). Third, infection may also induce RML through direct viral myositis or bacterial toxin-mediated muscle injury (Nuñez et al., 2024; Chenouard et al., 2018).

These considerations suggest that RML in AIS may represent a multifactorial syndrome arising from the interaction of immobilization, pharmacologic exposures (particularly statins), and infectious complications. Beyond etiologic complexity, RML could mark greater disease severity and adversely influence outcomes through downstream effects such as myoglobin-associated acute kidney injury (AKI), electrolyte disturbances, and systemic inflammation. However, despite these biologically plausible links, data delineating the clinical characteristics and prognostic implications of RML among hospitalized patients with AIS remain limited.

To address this gap, we conducted a 10-year, single-center retrospective study of hospitalized patients with AIS. The present study represents the first large-scale retrospective analysis aimed at elucidating the interplay between RML and AIS among hospitalized patients. By focusing on clinical characteristics, comorbidities, and in-hospital outcomes, we seek to provide evidence-based insights that inform treatment strategies and enhance patient prognosis. Our findings will contribute to the understanding of how RML may affect the clinical course of AIS, ultimately guiding clinicians in optimizing care for this vulnerable patient population. Our primary question was whether AIS patients who develop RML differ in baseline features, complications, and in-hospital outcomes from AIS patients who do not. Accordingly, we compared two groups drawn from the same source population of AIS admissions: an RML group defined by a validated CK-based threshold and an sex- and age-matched non-RML control group.

## Materials & Methods

A retrospective study was conducted on patients admitted to the Department of Neurology from January 2014 to December 2023. This study adhered to the principles outlined in the Declaration of Helsinki and was approved by the Ethics Committee of Shanghai Baoshan District Wusong Central Hospital (Project No. 2024-P-04). Upon review, the Committee determined that the retrospective design, use of de-identified records, and absence of any patient contact or intervention posed no more than minimal risk, and therefore waived the requirement for informed consent in accordance with the approved protocol.

The primary outcome was a composite of in-hospital mortality or DAMA. Outcomes were limited to the in-hospital period; standardized post-discharge endpoints (*e.g.*, 90-day modified Rankin Scale, 90-day mortality, readmissions, renal recovery) were not available in this dataset. Although DAMA is not a purely clinical endpoint, we included it because, in the Chinese healthcare context, it often signals clinical deterioration or a poor prognosis when families choose to take critically ill patients’ home. Secondary outcomes were the individual components (mortality and DAMA), reported separately. We extracted patient data from medical records, including medical history, laboratory test results, hospitalization costs, clinical outcomes, and other relevant information. For handling missing values in the dataset, we performed imputation using the group median before conducting further statistical analyses. RML was defined as a peak creatine kinase (CK) concentration >1,000 U/L, consistent with widely used thresholds and recommendations in the literature (Stahl, Rastelli & Schoser, 2020; Cabral et al., 2020; Chavez et al., 2016). To investigate the characteristics of patients with AIS combined with RML, we compared the RML group (AIS with RML, defined as peak CK >1,000 U/L) to the control group (AIS without RML, peak CK ≤ 1,000 U/L). We excluded patients with concurrent acute myocardial infarction (AMI). AMI independently elevates CK, especially CK-MB, and myoglobin. It also follows distinct diagnostic and management pathways. This situation risks misclassifying myocardial injury as RML and confounding the association between AIS-related RML and clinical outcomes (Aydin et al., 2019). Excluding AMI enhances cohort homogeneity and internal validity by focusing on skeletal muscle–derived CK elevations rather than cardiac sources (Stahl, Rastelli & Schoser, 2020; Chavez et al., 2016).

Controls were selected from AIS patients without RML using a reproducible, Python-based, stratified random sampling process. We targeted a 1:1 match to the RML group (CK > 1,000 U/L; no AMI). First, we stratified by sex to ensure identical composition (72.6% male, 27.4% female). Next, within each sex stratum, we used 5-year age bins, using the RML group’s age distribution as the template. The matching algorithm was implemented using Python (version 3.10.13) with a fixed random seed of 42 (using numpy.random.seed) to enable exact replication. The sampling procedure followed a hierarchical matching strategy. For each sex-age stratum in the RML group, we first attempted to randomly select the same number of patients from the identical stratum in the control pool. When insufficient patients were available in the exact age stratum, we employed a proximity-based selection from adjacent 5-year age intervals within the same gender category. This approach prioritized the closest available age matches while maintaining gender consistency. The algorithm continued iteratively through adjacent age strata (±5 years, ±10 years, *etc*.) until the required sample size was achieved for each sex-age combination. Matching quality was assessed by Student’s *t*-test for mean age (*p* = 0.912) and the Kolmogorov–Smirnov test for overall age distribution (*p* = 0.954). Because sex and age were prespecified matching variables, they are presented to document balance and were not treated as comparative findings in the results; hypothesis testing for these variables was not performed or interpreted. The final matched control group comprised 146 subjects with identical sex composition (106 males, 40 females) and comparable age characteristics (median age 80 years, IQR 69–85.25) to the RML group, ensuring adequate control for these potential confounding variables in subsequent analyses. CK, myoglobin, and troponin I were recorded at CK peak, while all other test indicators were taken from the first measurements after admission. The National Institutes of Health Stroke Scale (NIHSS) score was evaluated within 24 h post-admission (You et al., 2024).

Categorical data are presented as frequencies and percentages (n (%)). Non-normal distributions are summarized using the median and interquartile range (IQR). Statistical analysis was performed using the *χ*^2^ test for categorical data and the Wilcoxon test for continuous data that is not normally distributed. Patients were categorized into three prespecified strata: non-RML (CK ≤ 1,000 U/L), moderate RML (CK 1,001–4,999 U/L), and severe RML (CK ≥ 5,000 U/L). We compared the incidence of poor outcomes using the *χ*^2^ test and assessed trend using the Cochran–Armitage test. Because several CK strata were small—particularly the moderate-RML stratum (*n* = 12)—we did not fit multivariable models within the CK-stratified analyses to avoid overfitting and sparse-data bias. These comparisons are underpowered and should be considered exploratory and hypothesis-generating. For subgroup and interaction analyses involving multiple pairwise tests, we applied Bonferroni correction and FDR (Benjamini–Hochberg) adjustment. However, the number of subgroup and interaction tests may still increase the residual risk of type I error. Findings should be interpreted with caution, acknowledging potential confounding and the impact of multiple testing. We report Bonferroni-adjusted *p*-values for all pairwise comparisons.

The composite outcome (death or DAMA) served as the dependent variable. Variable selection followed a combined clinical and statistical strategy. First, established prognostic factors (NIHSS score > 15; diabetes; coronary heart disease) were forced into the model. Second, variables with univariate *p* < 0.10 were considered as candidates (infection, AKI, fall history, elevated troponin I, elevated creatinine, elevated CRP, and days from admission to CK peak > 2 days). To mitigate multicollinearity, when highly correlated variables were identified (*e.g.*, NIHSS score > 15 *vs.* consciousness disorder; overall infection *vs.* pulmonary infection), we retained the more standardized measure. The presence of RML (RML group) was specified a priori as the primary exposure. All selected variables were entered simultaneously. A *p*-value of less than 0.05 was considered statistically significant.

## Results

### Group classifications

**Figure 1:**
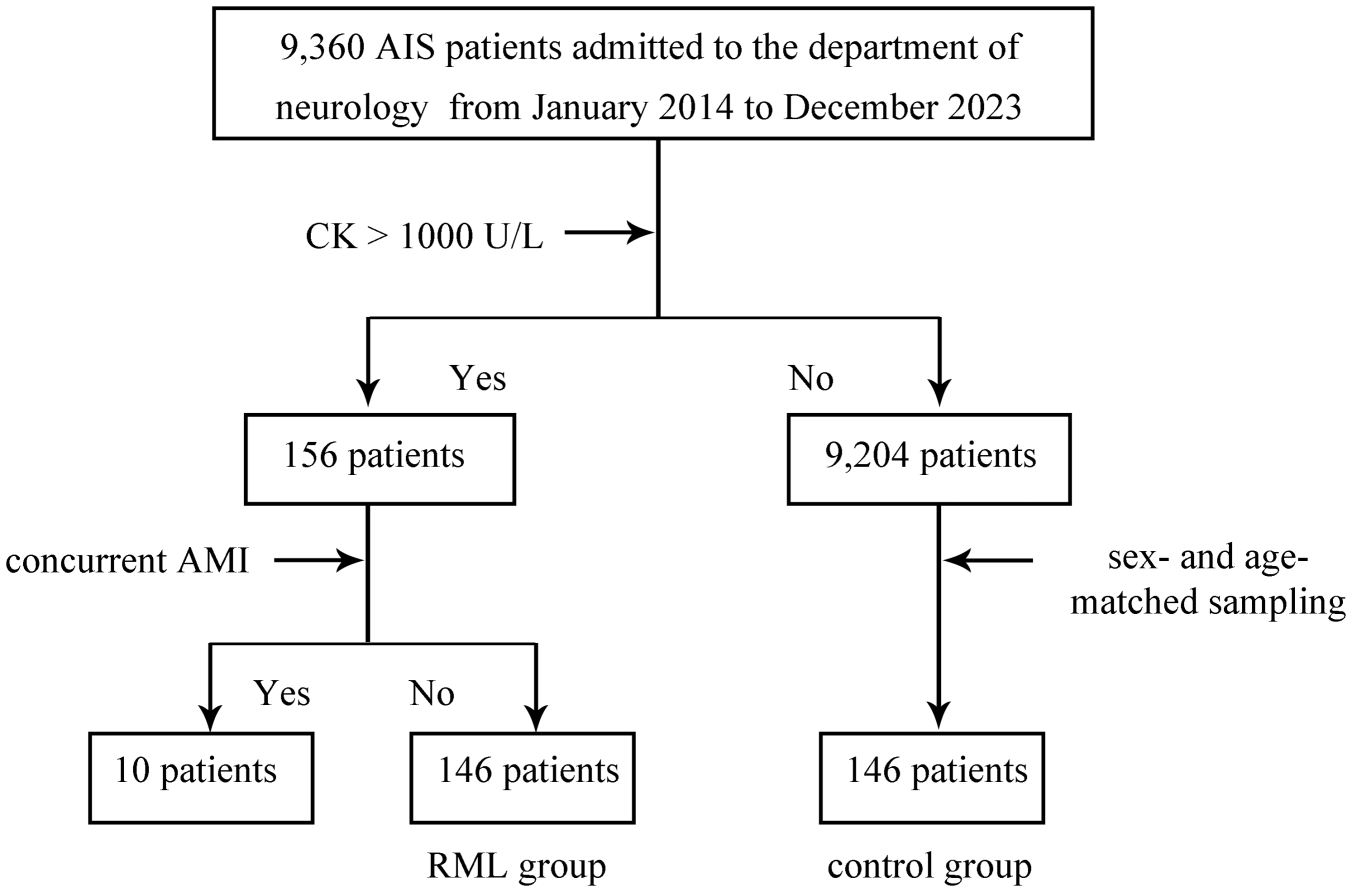
Study cohort selection and group assignment. Flow diagram for patients admitted with acute ischemic stroke (AIS) to the Department of Neurology between January 2014 and December 2023. Rhabdomyolysis (RML) was defined as peak creatine kinase (CK) > 1,000 U/L. Of 9,360 AIS patients, 156 met the CK criterion; 10 with concurrent acute myocardial infarction (AMI) were excluded, yielding 146 patients in the RML group. From the remaining 9,204 AIS patients with CK ≤ 1,000 U/L, 146 sex- and age-matched controls were selected by stratified random sampling. The diagram shows inclusion, exclusion, and final group sizes. Abbreviations: AIS, acute ischemic stroke; RML, rhabdomyolysis; CK, creatine kinase; AMI, acute myocardial infarction.

From January 2014 to December 2023, a total of 9,360 patients admitted to the Department of Neurology were confirmed to have AIS as their primary diagnosis (Fig. 1). A threshold of 1,000 U/L was used to diagnose RML based on CK levels. Of the 156 patients meeting this criterion, 10 presented with concurrent AMI and were excluded, leaving 146 patients in the RML group. Meanwhile, out of the remaining 9,204 AIS patients with CK levels ≤ 1,000 U/L, 146 sex- and age-matched individuals were selected as the control group. Consequently, two main study cohorts were established: a 146-patient RML group and a matched control group of 146 individuals.

### Patient characteristics

As shown in Table 1, there were no significant differences between the RML group and the Control group with respect to sex (72.60% *vs.* 72.60%, *p* = 1.000) and median age (80 [69–85] *vs.* 80 [69–85.25], *p* = 0.953). However, the RML group had a significantly higher prevalence of diabetes mellitus (43.15% *vs.* 26.03%, *p* = 0.002) and coronary heart disease (22.60% *vs.* 10.96%, *p* = 0.008). Additionally, patients in the RML group more frequently reported a history of falls (26.03% *vs.* 9.59%, *p* < 0.001), consciousness disorders (38.36% *vs.* 6.16%, *p* < 0.001), and had a greater proportion of moderate-to-severe NIHSS score (>15 points: 24.66% *vs.* 3.42%, *p* < 0.001).

**Table 1:** Comparison of characteristics between RML group and control group.

	Control group (*n* = 146)	RML group (*n* = 146)	*p* value
**Patient characteristics**			
Male	106 (72.60)	106 (72.60)	1.000
Age (years)	80 [69–85]	80 [69–85.25]	0.946
Hypertension	98 (67.12)	110 (75.34)	0.121
Diabetes	38 (26.03)	63 (43.15)	0.002
Coronary heart disease	16 (10.96)	33 (22.60)	0.008
Fall	14 (9.59)	38 (26.03)	<0.001
Consciousness disorder	9 (6.16)	56 (38.36)	<0.001
NIHSS score (>15)	5 (3.42)	36(24.66)	<0.001
**Clinical symptoms, complications, and the use of lipid-lowering drugs**			
Muscle pain	1 (0.68)	0 (0)	0.500
Muscle weakness	85 (58.22)	114 (78.08)	<0.001
Dark-colored urine	0 (0)	0 (0)	1.000
Infection	48 (32.88)	116 (79.45)	<0.001
Pulmonary infection	41 (28.08)	111 (76.03)	<0.001
Other	7 (4.79)	5 (3.42)	0.555
Acute kidney injury	8 (5.48)	53 (36.30)	<0.001
Lipid-lowering drugs	145 (99.32)	137 (93.84)	0.010
Atorvastatin (20mg/d or 40mg/d)	111 (76.03)	109 (74.66)	0.786
Double dose (40mg/d)	16 (10.96)	14 (9.59)	0.700
Other#	34 (23.29)	28 (19.18)	0.461
**Initial post-admission laboratory results**			
Hemoglobin (<11 g/dl)	20 (13.70)	25 (17.12)	0.418
C-reactive protein (>10 mg/L)	33 (22.60)	115 (78.77)	<0.001
Elevated creatinine	40 (27.40)	62 (42.47)	0.007
Albumin (<35 g/L)	46(31.50)	63(43.15)	0.040
Uric acid (>420 µmol/L)	31 (21.23)	39 (26.71)	0.273
Alanine Aminotransferase (>40 U/L)	7 (4.79)	25 (17.12)	<0.001
Cholesterol (>5.2 mmol/L)	33 (22.60)	27 (18.49)	0.385
Triglycerides (>1.7 mmol/L)	32(21.92)	22(15.07)	0.132
LDL-C (>3.4 mmol/L)	29 (19.86)	22 (15.07)	0.281
**Laboratory test results at the time of peak CK levels**			
Days from admission to peak CK (>2 days)	11 (7.53)	35 (23.97)	<0.001
Peak CK (U/L)	77 [53–117]	1,482.5 [1,160.0–2,790.5]	<0.001
Myoglobin (>1,000 ng/ml)	0 (0.00)	45 (30.82)	<0.001
Troponin I (>0.03 ng/ml)	22 (15.07)	92 (63.01)	<0.001

**Notes.**

Categorical data are expressed as frequencies and percentages (n (%)), while data with non-normal distributions are summarized as the median and interquartile range (IQR).

Abbreviations RML rhabdomyolysis ALT alanine aminotransferase NIHSS National Institutes of Health Stroke Scale CK creatine kinase Elevated creatinine serum creatinine >111 μmol/L in males; or >81 μmol/L in females; LDL-C Low-density lipoprotein cholesterol

Other#: refers to lipid-lowering drugs other than atorvastatin. Sex and age were used as matching variables, and the *p*-values were presented solely for demonstrating balance rather than for inferential interpretation.

### Comorbidities and laboratory findings

The incidence of infection was markedly higher in the RML group (79.45% *vs.* 32.88%, *p* < 0.001), particularly pulmonary infection (76.03% *vs.* 28.08%, *p* < 0.001). AKI occurred in 35.36% of the RML group, compared to 5.48% in controls (*p* < 0.001). Interestingly, lipid-lowering drug use was lower in the RML group than in the control group (93.84% *vs.* 99.32%, *p* = 0.010). However, there were no significant differences between the two groups in the utilization rate of atorvastatin (74.66% *vs.* 76.03%, *p* = 0.786) or in the use of double-dose atorvastatin (9.59% *vs.* 10.96%, *p* = 0.700). Regarding laboratory data, a significantly larger percentage of RML patients had elevated CRP levels (>10 mg/L: 78.77% *vs.* 22.60%, *p* < 0.001). Indicators of organ involvement, such as creatinine elevation (42.47% *vs.* 27.40%, *p* = 0.007), and hypoalbuminemia (43.15% *vs.* 31.50%, *p* = 0.040) were also more frequent in the RML group. Notably, peak CK levels were significantly higher in the RML group (median 1,482.5 [1,160.0–2,790.5] U/L *vs.* 77 [53–117] U/L, *p* < 0.001), with correspondingly elevated myoglobin (>1,000 ng/ml: 30.82% *vs.* 0%, *p* < 0.001) and troponin I (>0.03 ng/ml: 63.01% *vs.* 15.07%, *p* < 0.001).

### Clinical outcomes

Table 2 compares in-hospital outcomes. The RML group showed a significantly longer median hospital stay (15 [11–21.25] *vs.* 11 [9–14] days, *p* < 0.001) and higher median hospitalization costs (28.43 × 10^3^ (22.18 × 10^3^–49.82 × 10^3^) RMB *vs.* 19.51 × 10^3^ (16.16 × 10^3^–23.60 × 10^3^) RMB, *p* < 0.001). Mortality in the RML group was substantially greater (18.49% *vs.* 0.68%, *p* < 0.001), and the incidence of DAMA was also higher (10.27% *vs.* 2.74%, *p* = 0.009). The poor outcome rate, defined as the sum of mortality and DAMA, was significantly higher in the RML group than in the control group (28.77% *vs.* 3.42%, *p* < 0.001). Taken together, these findings suggest that AIS patients with RML exhibit more severe clinical manifestations and experience worse in-hospital outcomes than their counterparts without RML.

**Table 2:** Outcome of AIS patients without and with rhabdomyolysis.

	Control group(*n* = 146)	RML group (*n* = 146)	*p* value
Hospital stay (days)	11 (9–14)	15 (11–21.25)	<0.001
Hospitalization costs (×10^3^ RMB)*	19.51 [16.16–23.60]	28.43 [22.18–49.82]	<0.001
Mortality#	1 (0.68)	27 (18.49)	<0.001
DAMA †	4 (2.74)	15 (10.27)	0.009
Poor outcome ‡	5 (3.42)	42 (28.77)	<0.001

**Notes.**

Categorical data are expressed as frequencies and percentages (n (%)), while data with non-normal distributions are summarized as the median and interquartile range (IQR). Mortality#: all-cause in-hospital mortality. DAMA†:Discharge against medical advice (family ended treatment due to the patient’s critical condition and lack of improvement, resulting in automatic discharge). Poor outcome‡: defined as the combined incidence of death or DAMA.

### CK-stratified exploratory outcomes

The rates of POR differed across the three CK-defined groups: 3.4% (5/146) in the non-RML group (CK ≤ 1,000 U/L), 41.7% (5/12) in the moderate RML group (CK 1,001–4,999 U/L), and 27.6% (37/134) in the severe RML group (CK ≥ 5,000 U/L). Pairwise comparisons showed that both the moderate RML (Bonferroni-adjusted *p* < 0.001) and severe RML (Bonferroni-adjusted *p* < 0.001) groups had significantly higher rates of POR than the non-RML group. However, the difference between the moderate and severe RML groups was not statistically significant (Bonferroni-adjusted *p* = 0.98). Precision is limited, especially in the moderate-RML stratum (*n* = 12), leading to imprecise estimates. Despite Bonferroni correction for pairwise comparisons, the multiplicity of subgroup and interaction tests may still increase the risk of type I error. These findings are hypothesis-generating and should be interpreted with caution.

### Multifactor logistic analysis of factors associated with poor outcome in AIS patients

**Figure 2:**
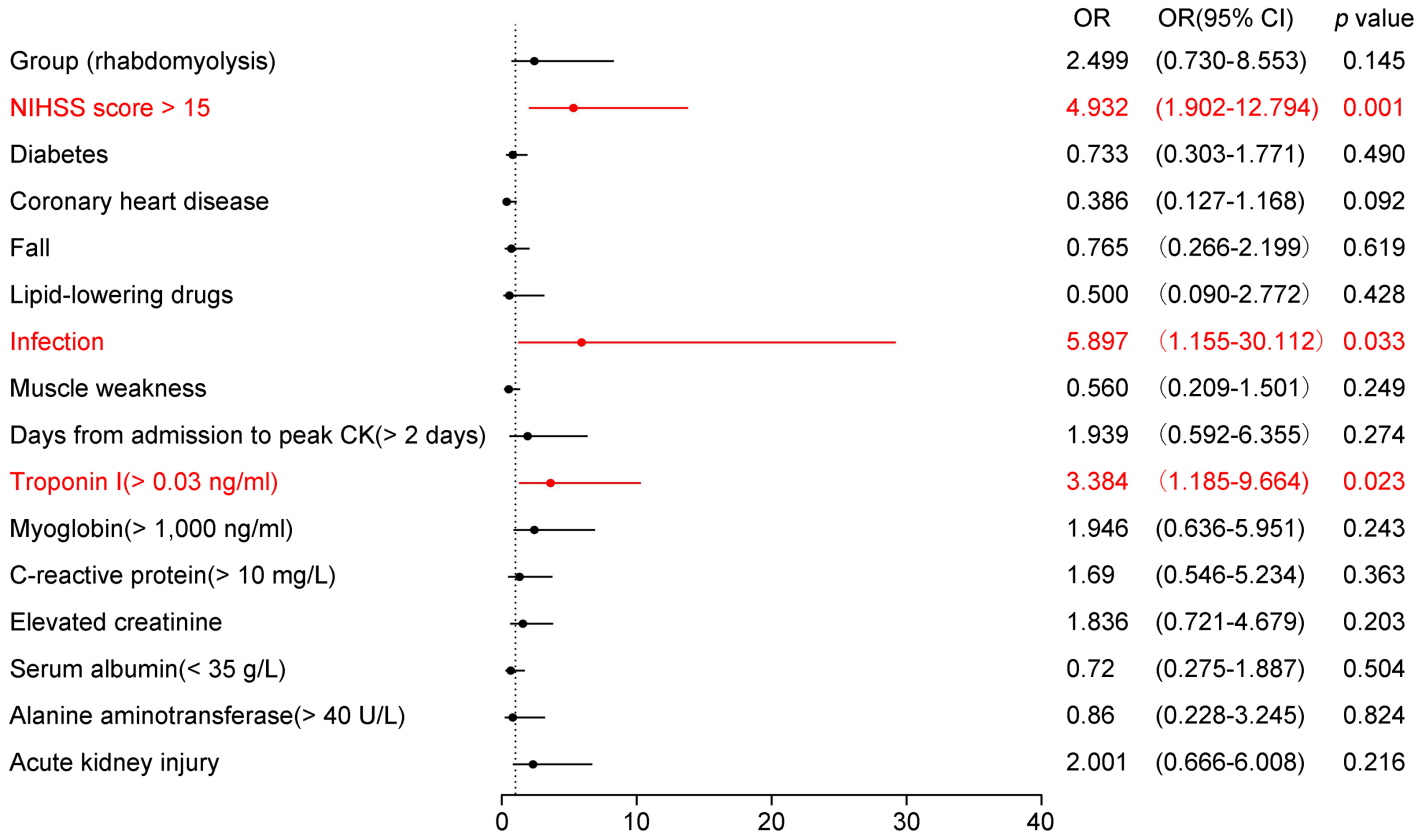
Forest plot of adjusted odds ratios for poor in-hospital outcome in acute ischemic stroke (AIS). Points show adjusted odds ratios (OR) with 95% confidence intervals (CI) from a multivariable logistic regression; the vertical dashed line marks OR = 1. ORs are plotted on a log scale. Significant predictors (*P* < 0.05) are highlighted. The model adjusted for: NIHSS > 15, diabetes, coronary heart disease, infection, history of falls, elevated troponin I (> 0.03 ng/mL), elevated creatinine, elevated C-reactive protein (> 10 mg/L), hypoalbuminemia (< 35 g/L), alanine aminotransferase > 40 U/L, acute kidney injury, days from admission to CK peak > 2 days, lipid-lowering drugs, and rhabdomyolysis (yes/no). Binary predictors were coded as present *vs* absent (reference = absent); for NIHSS the reference was ≤ 15; for RML the reference was non - RML. Model sample . Abbreviations: AIS, acute ischemic stroke; RML, rhabdomyolysis; OR, odds ratio; CI, confidence interval; NIHSS, National Institutes of Health Stroke Scale.

In the multivariable logistic regression model, the pre-specified predictors (NIHSS score > 15, diabetes, coronary heart disease), the candidate variables with univariate *p* < 0.10 (infection, AKI, fall history, elevated troponin I, elevated creatinine, elevated CRP, and days from admission to peak CK > 2 days), and the primary exposure (RML group) were included. Due to collinearity, consciousness disorder and pulmonary infection were not entered, with NIHSS score > 15 and overall infection retained, respectively (see Methods for details). Since the RML group variable demonstrated greater significance than peak CK, we included the group variable instead of peak CK in the multivariable logistic regression analysis. Figure 2 presents the results of the multivariable logistic regression analysis examining potential predictors of unfavorable outcomes in patients with AIS. Among the variables assessed, three reached statistical significance (highlighted in red). An NIHSS score > 15 (OR = 4.932, 95% CI [1.902–12.794], *p* = 0.001) was strongly associated with poor outcomes, indicating that severe baseline neurological deficits substantially heighten risk. Additionally, infection (OR = 5.897, 95% CI [1.550–30.112], *p* = 0.033) emerged as a significant complication predisposing patients to worse prognosis. Elevated troponin I levels (>0.03 ng/ml, OR = 3.384, 95% CI [1.185–9.664], *p* = 0.023) were also linked to an unfavorable outcome, reflecting the cardio-neurological interplay in AIS. Other variables, such as diabetes, coronary heart disease, history of falls, lipid-lowering drug use, muscle weakness, myoglobin >1,000 ng/ml, elevated CRP, creatinine, hypoalbuminemia, alanine aminotransferase >40 U/L, and AKI, did not reach statistical significance in this model. Notably, the presence or absence of RML was not identified as an independent risk factor for poor prognosis in AIS patients.

### Subgroup analysis of risk factors for poor outcomes in AIS patients

**Figure 3:**
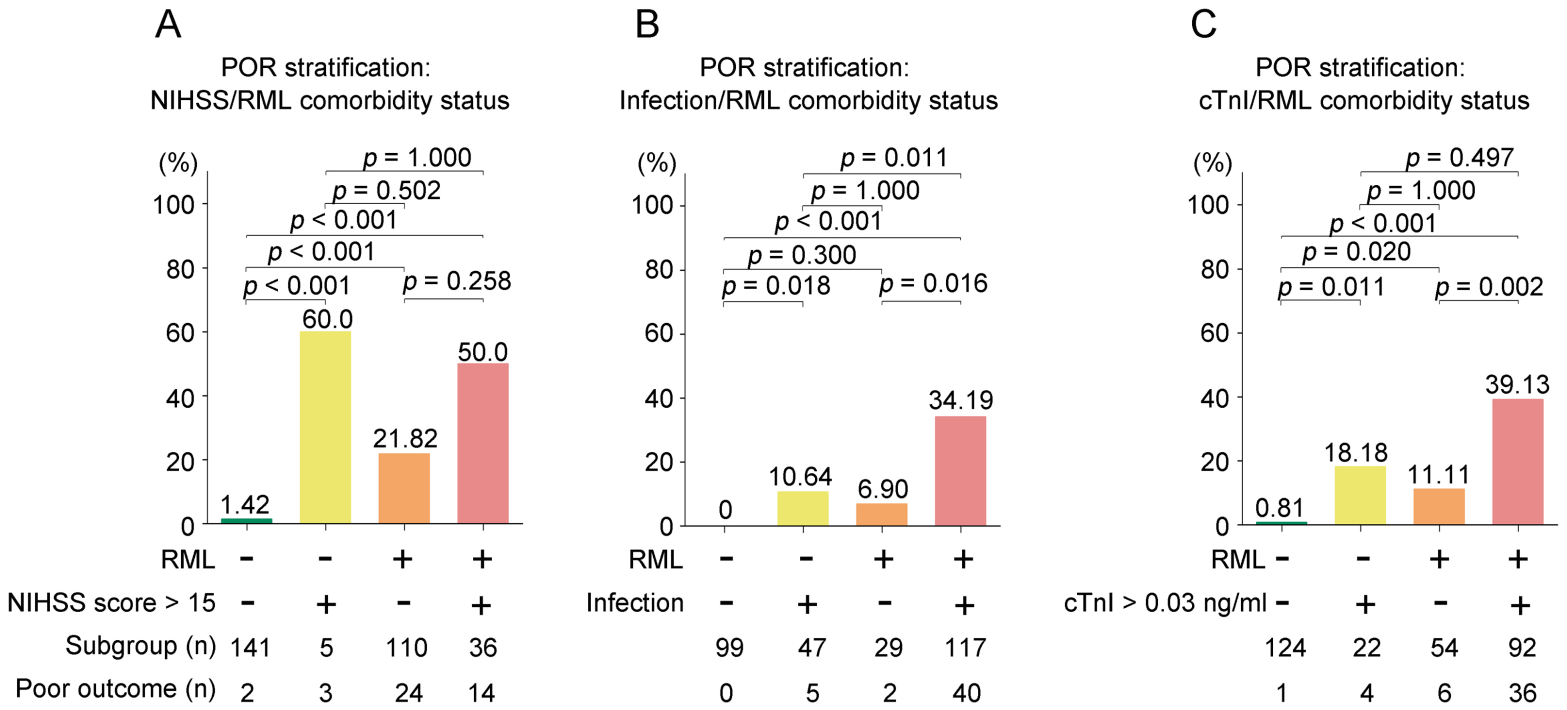
Subgroup analyses of poor in-hospital outcome (POR: death or discharge against medical advice, DAMA) by RML and comorbidity status. Bars show the proportion with POR; numbers beneath bars indicate subgroup size and number with POR. (A) Stratification by NIHSS < 15; (B) stratification by infection; (C) stratification by elevated cardiac troponin I (cTnI < 0.03 ng/mL). Within each panel, bars are ordered as: comorbidity(−)/RML(−), comorbidity(+)/RML(−), comorbidity(−)/RML(+), comorbidity(+)/RML(+). Pairwise comparisons used (*χ*^2^/Fisher’s exact) tests; *P* values above bars are Bonferroni-adjusted). Abbreviations: RML, rhabdomyolysis; POR, poor outcome; DAMA, discharge against medical advice; NIHSS, National Institutes of Health Stroke Scale; cTnI, cardiac troponin I; AIS, acute ischemic stroke.

Figure 3 presents a subgroup analysis stratified by the presence of RML alongside three key risk factors: NIHSS score > 15 (Fig. 3A), infection (Fig. 3B), and elevated troponin I (>0.03 ng/ml) (Fig. 3C). All pairwise comparisons were adjusted for multiple testing using both Bonferroni correction and FDR (Benjamini–Hochberg) method.

For RML/NIHSS score > 15 stratification (Fig. 3A), patients with both RML and NIHSS score > 15 demonstrated the highest incidence of adverse outcomes (50.0%), followed by RML alone (21.82%) and neither risk factor (1.42%). After multiple comparison adjustment, the differences between RML+NIHSS > 15 *vs.* neither factor remained significant (Bonferroni *p* < 0.001, FDR *p* < 0.001), as did RML alone *vs.* neither factor (Bonferroni *p* < 0.001, FDR *p* < 0.001). The difference in adverse outcome rates between the RML+NIHSS > 15 and the NIHSS > 15 alone groups was not statistically significant (*p* = 0.084); however, the NIHSS > 15 alone subgroup was very small (*n* = 5), rendering the comparison underpowered and the null result more likely due to low statistical power rather than a true absence of effect.

For RML/infection stratification (Fig. 3B), the highest poor outcome rate was observed in patients with both RML and infection (34.19%), compared to infection alone (10.64%), RML alone (6.90%), and neither condition (0%). After correction, RML+infection *vs.* neither factor (Bonferroni *p* < 0.001, FDR *p* < 0.001) and RML+infection *vs.* infection alone (Bonferroni *p* = 0.018, FDR *p* = 0.011) remained statistically significant.

For RML/troponin I stratification (Fig. 3C), patients with both RML and elevated troponin I exhibited the highest rate of adverse outcomes (39.13%), exceeding elevated troponin I alone (18.18%), RML alone (11.11%), and neither factor (0.81%). Following multiple comparison adjustment, RML+elevated troponin I *vs.* neither factor (Bonferroni *p* < 0.001, FDR *p* < 0.001) and RML+elevated troponin I *vs.* elevated troponin I alone (Bonferroni *p* = 0.020, FDR *p* = 0.011) remained significant. These subgroup comparisons are unadjusted (no covariate adjustment), underpowered, and hypothesis-generating; they do not establish independent or causal effects of RML.

## Discussion

The co-occurrence of AIS and RML has been noted in previous case reports (Hsu, Chiu & Liao, 2009; Kim et al., 2023; Roebroek & Korten, 1996). However, their combined presence in inpatient neurology settings has not been specifically examined. To the best of our knowledge, this is the first large-scale retrospective study to investigate the interplay between these two conditions in hospitalized neurology patients. Our results demonstrate that patients with AIS who develop RML exhibit increased clinical severity and worse in-hospital outcomes compared to their counterparts without RML. Our analysis revealed that 1.56% (146/9,360) of AIS patients met the diagnostic criteria for RML. After sex- and age-matching and multivariable adjustment for measured covariates, the crude differences between RML and non-RML groups were largely explained by stroke severity, infection, and myocardial injury. RML may mark systemic severity rather than exert an independent effect. Consistent with this interpretation, we do not propose a shared primary pathophysiology between AIS and RML; rather, we view RML as arising from secondary mechanisms triggered by AIS (immobilization/pressure, infection, pharmacologic exposures). Residual and unmeasured confounding cannot be excluded.

In exploratory CK stratified sensitivity analyses, unadjusted rates of poor outcomes were higher in both the moderate RML (CK 1,001–4,999 U/L) and severe RML (CK ≥ 5,000 U/L) strata than in the non-RML group, whereas the difference between the moderate RML and severe RML strata was not statistically significant. This pattern suggests that RML itself, rather than additional CK elevation beyond 5,000 U/L, may be the principal signal of risk. Given the small sample size in the moderate RML stratum and the unadjusted nature of these comparisons, these findings should be interpreted cautiously and viewed as supportive of, but not definitive beyond, our main results.

The pathophysiological links between AIS and RML in our cohort are consistent with established mechanisms and are supported by our data. First, markers of severe stroke and reduced vigilance in the RML group, namely consciousness disorders (38.36%) and NIHSS score >15 (24.66%), support the immobilization/compression hypothesis, as these patients are at increased risk of prolonged bed rest and local muscle pressure. Notably, impaired consciousness at stroke onset—particularly in large hemispheric infarction—is associated with older age, atrial fibrillation, and higher baseline NIHSS scores. It also portends malignant cerebral edema, pneumonia, and worse outcomes. However, RML is not consistently highlighted as a common complication in this context, and a direct, independent association with NIHSS severity has not been demonstrated (Paternostro et al., 2021; Zöllner et al., 2020). Second, the higher prevalence of falls in the RML group (26.03% *vs* 9.59%, *p* < 0.001) suggests that trauma-related muscle injury contributes to RML (Morin, Somme & Corvol, 2024). Third, while atorvastatin use was similar between groups (74.66% *vs* 76.03%, *p* = 0.786), overall lipid-lowering therapy was paradoxically lower among RML patients (93.84% *vs* 99.32%, *p* = 0.010), possibly reflecting clinician awareness and early discontinuation after CK elevation or concern for intolerance (Oberhoffer et al., 2025; Tournadre, 2020). Finally, infection were strikingly prevalent in the RML group (overall 79.45%; pulmonary 76.03%). This is consistent with infection-mediated pathways in which pneumonia can trigger RML through direct invasion, endotoxin release, and cytokine-driven myotoxicity (Chenouard et al., 2018; Solhjou et al., 2020; Geng et al., 2021). Taken together, these findings suggest that RML in AIS often arises from multifactorial processes, including immobilization and trauma, pharmacologic exposures, and infection, and may function more as a marker of systemic severity than as an independent driver of poor outcomes, consistent with our multivariable results.

The RML group exhibited a higher burden of complications, including diabetes, coronary heart disease, and a history of falls, as well as acute indicators such as delirium and severe NIHSS scores. These findings align with previous evidence suggesting that long-term immobilization, metabolic stress, and systemic inflammation are associated with muscle injury in critically ill patients (Al-Azzawi, Razak & Hammady, 2019; Jin & Tong, 2020; Brnčić et al., 2002; Sakai et al., 2020). The significant prevalence of infection (particularly pulmonary infection) and AKI in the RML group underscores the systemic impact of skeletal muscle injury, which may be driven by myoglobin toxicity and inflammatory cascade reactions (Solhjou et al., 2020; Lee & Ahn, 2022). Interestingly, while the use of atorvastatin was comparable between the two groups, the frequency of lipid-lowering therapy was lower in RML patients. This suggests potential under-prescription or confounding contraindications (*e.g.*, liver or kidney impairment) in this high-risk cohort.

Our multivariable analysis identified three independent predictors of poor outcomes: an NIHSS score > 15 (OR = 4.932), infection (OR = 5.897), and elevated troponin I (OR = 3.384). The National Institutes of Health Stroke Scale (NIHSS) is a critical tool for assessing stroke severity and predicting patient outcomes. An NIHSS score > 15 strongly predicts a poor prognosis, indicating a high likelihood of death or severe disability within 90 days after a stroke (Adams et al., 1999). Our study revealed a significant increase in the rate of NIHSS scores greater than 15 in the RML group, reaching 24.66%. Further subgroup analysis indicated that AIS patients with RML and NIHSS scores above 15 had a poor prognosis rate of 50%.

While RML is frequently associated with physical trauma, intense exercise, or drug use, it may also arise from a variety of infection (Buckholz, 2020; Samardzic et al., 2024; Dell & Schulman, 1997; Koubar et al., 2017). In a retrospective study conducted in pediatric emergency departments, 59.46% patients had infection (Chen et al., 2013). A retrospective study conducted by Johns Hopkins Hospital revealed that 1.47% of cases were attributed to infection (Melli, Chaudhry & Cornblath, 2005). In our patients with RML, the incidence of coinfection was 79.45%. Our logistic regression analysis identified infection as a significant risk factor for developing RML in AIS patients. Infection can trigger RML through several mechanisms, including direct muscle damage caused by viruses, toxin release from bacteria such as Legionella and Staphylococcus, and systemic inflammation that leads to tissue hypoxia (Huerta-Alardín, Varon & Marik, 2005). Furthermore, factors like fever, dehydration, and electrolyte imbalances can indirectly contribute to the development of RML (Same et al., 2019). In our study of AIS patients with RML, CRP elevation was observed in 78.77% of RML cases. This finding is consistent with the higher incidence of co-infection within this patient group. Accumulating evidence demonstrates that CRP plays a fundamental role in inflammatory processes and the host’s response to infection. This role encompasses modulation of the complement pathway, apoptosis, phagocytosis, nitric oxide (NO) release, and cytokine production, particularly interleukin-6 and tumor necrosis factor alpha (Sproston & Ashworth, 2018).

Subgroup analyses demonstrated significant synergistic effects between RML and other clinical factors, indicating a bidirectional relationship. This interaction was particularly evident when RML coexisted with elevated troponin I, with adverse outcomes markedly increasing (39.13% *versus* 18.18% with elevated troponin I alone), thereby highlighting the key role of the cardio-neurological axis in stroke pathophysiology. The cardio-neurological axis is important in understanding stroke, highlighting the complex connections between the brain and heart. Key factors include the hypothalamic-pituitary-adrenal (HPA) axis and sympathetic hyperactivity, which link stroke to heart issues (Fan et al., 2024). These factors can lead to neurogenic stress cardiomyopathy and other heart problems after brain injury. Inflammatory and immune responses also play a significant role in how the brain-heart axis functions after a stroke (Chen, Gu & Zhang, 2024). These responses can damage the heart further and are linked to worse outcomes. Additionally, new research suggests that gut dysbiosis may affect the brain-heart axis, influencing cardiovascular outcomes after a stroke (Battaglini et al., 2020).

The advantages of our study include its status as the first large-scale retrospective analysis investigating the interaction between RML and AIS in hospitalized neurological patients. We implemented strict 1:1 matching and thoroughly adjusted for confounding factors, yielding new insights into the synergistic effect of RML on stroke severity and systemic complications. By sampling cases and controls from the same AIS admission population, matching on sex and age, and explicitly excluding AMI to prevent CK-based misclassification, the group selection strategy strengthens internal validity for estimating associations between RML and in-hospital outcomes.

However, the retrospective, single-center design precludes causal inference and limits generalizability. Outcomes were restricted to the in-hospital period. We lacked standardized post-discharge endpoints (*e.g.*, 90-day modified Rankin Scale, 90-day mortality, readmissions, renal recovery). Therefore, our results primarily describe the in-hospital course and resource use. Including DAMA reflects local practice but is not a validated functional outcome and may misclassify prognosis. Longer-term implications should be interpreted with caution. Residual confounding (*e.g.*, frailty, immobilization duration, premorbid disability) and potential mediation (*e.g.*, *via* acute kidney injury or systemic inflammation) may bias estimates. Several subgroup strata were small (*e.g.*, moderate RML *n* = 12; NIHSS score >15 without RML *n* = 5), leading to underpowering and imprecise estimates. Moreover, the multiplicity of subgroup and pairwise comparisons may inflate the risk of type I error despite Bonferroni/FDR adjustments. Accordingly, subgroup results should be regarded as exploratory and hypothesis-generating and interpreted with caution in light of potential residual confounding (*e.g.*, frailty, immobilization duration, premorbid disability) and possible mediation (*e.g.*, *via* AKI or systemic inflammation).

## Conclusions

This study is the first large-scale retrospective analysis examining the co-occurrence of RML and AIS through secondary pathways in hospitalized neurological patients. Our findings reveal that patients in the RML group have higher rates of comorbidities, acute complications, and prolonged hospital stays, along with a significantly increased mortality rate. Although RML was not identified as an independent predictor of poor outcome, its interaction with NIHSS score > 15, infection, and elevated troponin I levels substantially heightens the risk of poor prognosis. These results emphasize the critical need for close monitoring of RML and associated complications in AIS patients, along with comprehensive management strategies to mitigate the elevated risk of adverse outcomes in this vulnerable population. Overall, our findings support RML as a marker of systemic severity mediated by secondary mechanisms precipitated by AIS, rather than a random co-morbidity or a direct consequence of the primary ischemic pathology. Future research should focus on further elucidating the mechanisms underlying this interaction and exploring therapeutic interventions that could enhance patient outcomes. Because post-discharge functional outcomes were unavailable, our conclusions apply to the in-hospital period; confirmation with standardized 90-day follow-up is warranted.

## Supplemental Information

10.7717/peerj.20645/supp-1 Supplemental Information 1 Data dictionary CRP: C-reactive protein. LDL-C : Low-density lipoprotein cholesterol.

10.7717/peerj.20645/supp-2 Supplemental Information 2 Missing proportion of each variable and imputation rules CRP: C-reactive protein. LDL-C : Low-density lipoprotein cholesterol.

10.7717/peerj.20645/supp-3 Supplemental Information 3 Clinical, Laboratory, and Outcome Characteristics in the Rhabdomyolysis and Control Groups RML: Stands for Rhabdomyolysis Group. Ctrl: Represents the control group. N: Represents the absence of original data.

## Additional Information and Declarations

### Data Availability

The following information was supplied regarding data availability: The raw measurements are available in the Supplementary Files.

## References

[ref-1] Adams H, Davis P, Leira E, Chang K, Bendixen B, Clarke W, Woolson R, Hansen M (1999). Baseline NIH stroke scale score strongly predicts outcome after stroke. Neurology.

[ref-2] Al-Azzawi OFN, Razak MKA, Al Hammady SJ (2019). Rhabdomyolysis; is it an overlooked DKA complication. Diabetes & Metabolic Syndrome.

[ref-3] Aydin S, Ugur K, Aydin S, Sahin İ, Yardim M (2019). Biomarkers in acute myocardial infarction: current perspectives. Vascular Health and Risk Management.

[ref-4] Battaglini D, Robba C, Lopes da Silva A, Dos Santos Samary C, Leme Silva P, Dal Pizzol F, Pelosi P, Rocco PRM (2020). Brain–heart interaction after acute ischemic stroke. Critical Care.

[ref-5] Brnčić N, Višković I, Sasso A, Kraus I, Zamolo G (2002). Salmonella infection-associated acute rhabdomyolysis. Some pathogenic considerations. Archives of Medical Research.

[ref-6] Buckholz AP (2020). Clinical characteristics, and diagnosis, and outcomes of 6 patients with COVID-19 infection and rhabdomyolysis. Mayo Clinic Proceedings.

[ref-7] Cabral BMI, Edding SN, Portocarrero JP, Lerma EV (2020). Rhabdomyolysis. Disease-a-Month.

[ref-8] Chavez LO, Leon M, Einav S, Varon J (2016). Beyond muscle destruction: a systematic review of rhabdomyolysis for clinical practice. Critical Care.

[ref-9] Chen X, Gu J, Zhang X (2024). Brain-heart axis and the inflammatory response: connecting stroke and cardiac dysfunction. Cardiology.

[ref-10] Chen CY, Lin YR, Zhao LL, Yang WC, Chang YJ, Wu KH, Wu HP (2013). Clinical spectrum of rhabdomyolysis presented to pediatric emergency department. BMC Pediatrics.

[ref-11] Chenouard A, Travert B, Kuster A, De Lonlay P, Bourgoin P (2018). Virus or bacteria: is it the only cause of sepsis-induced rhabdomyolysis?. Pediatric Critical Care Medicine.

[ref-12] Dell KM, Schulman SL (1997). Rhabdomyolysis and acute renal failure in a child with influenza A infection. Pediatric Nephrology.

[ref-13] Fan X, Cao J, Li M, Zhang D, El-Battrawy I, Chen G, Zhou X, Yang G, Akin I (2024). Stroke related brain–heart crosstalk: pathophysiology, clinical implications, and underlying mechanisms. Advanced Science.

[ref-14] Geng Y, Qiang, Du Y-S, Peng N, Yang T-T, Zhang S-Y, Wu F-F, Lin H, Su L (2021). Rhabdomyolysis is associated with in-hospital mortality in patients with COVID-19. Shock.

[ref-15] Herpich F, Rincon F (2020). Management of acute ischemic stroke. Critical Care Medicine.

[ref-16] Hsu WY, Chiu NY, Liao YC (2009). Rhabdomyolysis and brain ischemic stroke in a heroin-dependent male under methadone maintenance therapy. Acta Psychiatrica Scandinavica.

[ref-17] Huerta-Alardín AL, Varon J, Marik PE (2005). Bench-to-bedside review: rhabdomyolysis—an overview for clinicians. Critical Care.

[ref-18] Jalal N, Gracies JM, Zidi M (2020). Mechanical and microstructural changes of skeletal muscle following immobilization and/or stroke. Biomechanics and Modeling in Mechanobiology.

[ref-19] Jin M, Tong Q (2020). Rhabdomyolysis as potential late complication associated with COVID-19. Emerging Infectious Diseases.

[ref-20] Kim S, Choi S, Nah S, Han SS (2023). Multiple cerebral infarctions and rhabdomyolysis after sildenafil citrate (Viagra^®^) intoxication: a case report. Journal of Emergency Medicine.

[ref-21] Koubar SH, Estrella MM, Warrier R, Moore RD, Lucas GM, Atta MG, Fine DM (2017). Rhabdomyolysis in an HIV cohort: epidemiology, causes and outcomes. BMC Nephrology.

[ref-22] Lee IH, Ahn DJ (2022). Rhabdomyolysis and acute kidney injury associated with salmonella infection: a report of 2 cases. The American Journal of Case Reports.

[ref-23] Melli G, Chaudhry V, Cornblath DR (2005). Rhabdomyolysis: an evaluation of 475 hospitalized patients. Medicine.

[ref-24] Montastruc J (2023). Rhabdomyolysis and statins: a pharmacovigilance comparative study between statins. British Journal of Clinical Pharmacology.

[ref-25] Morin A-G, Somme D, Corvol A (2024). Rhabdomyolysis in older adults: outcomes and prognostic factors. BMC Geriatrics.

[ref-26] Nuñez VG, De Almeida LV, Da Silva AC, Oliveira IDT, Martins NT, De Sousa Almeida YC (2024). Rhabdomyolysis secondary to influenza virus infection. Infections in Evidence.

[ref-27] Oberhoffer F, Rieger E, Schenk S, Hauer J, Chmiel R, Steinhauser M (2025). Statin-associated rhabdomyolysis: an exemplary case report and a mini-review of therapeutic management. Translational Pediatrics.

[ref-28] Paternostro C, Gopp L, Tomschik M, Krenn M, Weng R, Bointner K, Jäger F, Zulehner G, Rath J, Berger T, Zimprich F, Cetin H (2021). Incidence and clinical spectrum of rhabdomyolysis in general neurology: a retrospective cohort study. Neuromuscular Disorders.

[ref-29] Phipps MS, Cronin CA (2020). Management of acute ischemic stroke. Bmj.

[ref-30] Roebroek RM, Korten JJ (1996). Epileptic insults, cerebral infarction and rhabdomyolysis as complications of amphetamine use. Nederlands Tijdscrift Voor Geneeskunde.

[ref-31] Sakai K, Omizo H, Togashi R, Hayama Y, Ueno M, Tomomitsu Y, Nemoto Y, Asakawa S, Nagura M, Arai S, Yamazaki O, Tamura Y, Uchida S, Shibata S, Fujigaki Y (2020). Rhabdomyolysis-induced acute kidney injury requiring hemodialysis after a prolonged immobilization at home in 2 morbidly obese women: case reports with literature review. Renal Replacement Therapy.

[ref-32] Samardzic T, Muradashvili T, Guirguis S, Felek S, Pan SC, Tiyyagura S, Feinn R (2024). Relationship between rhabdomyolysis and SARS-CoV-2 disease severity. Cureus.

[ref-33] Same RG, McAleese S, Agwu AL, Arav-Boger R (2019). Acute HIV in an adolescent male with fever and rhabdomyolysis. Journal of Adolescent Health.

[ref-34] Solhjou Z, Castillo IP, Mikhael B, Chedid N, Barkoudah E, Sheridan A (2020). Rhabdomyolysis in SARS-CoV-2 infection. Journal of the American Society of Nephrology.

[ref-35] Sproston NR, Ashworth JJ (2018). Role of C-reactive protein at sites of inflammation and infection. Frontiers in Immunology.

[ref-36] Stahl K, Rastelli E, Schoser B (2020). A systematic review on the definition of rhabdomyolysis. Journal of Neurology.

[ref-37] Tournadre A (2020). Statins, and myalgia, and rhabdomyolysis. Joint Bone Spine.

[ref-38] You S, Wang Y, Wang X, Maeda T, Ouyang M, Han Q, Li Q, Song L, Zhao Y, Chen C, Delcourt C, Ren X, Carcel C, Zhou Z, Cao Y, Liu CF, Zheng D, Arima H, Robinson TG, Chen X, Lindley RI, Chalmers J, Anderson CS (2024). Twenty-four-hour post-thrombolysis NIHSS score as the strongest prognostic predictor after acute ischemic stroke: ENCHANTED study. Journal of the American Heart Association.

[ref-39] Zöllner J, Misselwitz B, Kaps M, Stein M, Konczalla J, Roth C, Krakow K, Steinmetz H, Rosenow F, Strzelczyk A (2020). National Institutes of Health Stroke Scale (NIHSS) on admission predicts acute symptomatic seizure risk in ischemic stroke: a population-based study involving 135,117 cases. Scientific Reports.

