# Supplementary material for: Characteristics and outcome of rhabdomyolysis in acute ischemic stroke patients: a 10-year retrospective study
Source: PeerJ. 2026 Jan 20;14:e20645. doi: 10.7717/peerj.20645 (PMC12829463; doi:10.7717/peerj.20645)
Supplement: Supplemental Information 1 — CRP: C-reactive protein. LDL-C : Low-density lipoprotein cholesterol. [file peerj-14-20645-s001.docx]

**Supplementary table S1** (data dictionary)

| Variable | Definition | Timing | Unit | Coding/Scale | Source |
| --- | --- | --- | --- | --- | --- |
| RML | Peak CK > 1,000 U/L | Peak CK timepoint | U/L | Binary (0/1) | EMR/lab |
| Poor outcome | In-hospital mortality or discharge against medical advice | Discharge | — | Binary (0/1) | EMR |
| Troponin I elevated | Troponin I > 0.03 ng/ml | Peak CK timepoint | ng/ml | Binary (0/1) | Lab |
| CRP elevated | CRP > 10 mg/L | First measurement after admission | mg/L | Binary (0/1) | Lab |
| ALT elevated | ALT > 40 U/L | First measurement after admission | U/L | Binary (0/1) | Lab |
| Infection | Physician-documented infection | In-hospital | — | Binary (0/1) | EMR |
| Pulmonary infection | Physician-documented pulmonary infection | In-hospital | — | Binary (0/1) | EMR |
| AKI | Physician-documented AKI; consistent with standard creatinine-based criteria where serial labs permit | In-hospital | — | Binary (0/1) | EMR/Lab |
| Hypoalbuminemia | Albumin below institutional lower reference limit | First measurement after admission | g/L | Binary (0/1) | Lab |
| Creatinine elevation | ＞ 111 μmol/L in males, ＞ 81 μmol/L in females | First measurement after admission | µmol/L | Binary (0/1) | Lab |
| Myoglobin elevated | Myoglobin > 1,000 ng/ml | Peak CK timepoint | ng/ml | Binary (0/1) | Lab |
| NIHSS > 15 | NIHSS assessed within 24 h of admission | 24 h post-admission | points | Binary (0/1) | EMR |
| Days to peak CK > 2 | Days from admission to peak CK > 2 | Derived | days | Binary (0/1) | Derived |

Binary (0/1)：1=Yes/Present，0=No/Absent
