# Supplementary material for: Characteristics and outcome of rhabdomyolysis in acute ischemic stroke patients: a 10-year retrospective study
Source: PeerJ. 2026 Jan 20;14:e20645. doi: 10.7717/peerj.20645 (PMC12829463; doi:10.7717/peerj.20645)
Supplement: Supplemental Information 2 — CRP: C-reactive protein. LDL-C : Low-density lipoprotein cholesterol. [file peerj-14-20645-s002.docx]

**Supplementary table S2** (missing proportion of each variable and imputation rules)

| Variable | Control group | RML group | total | Missing  (%) | Imputation/Handling |
| --- | --- | --- | --- | --- | --- |
| NIHSS | 10 | 3 | 13 | 4.45 | Within‑group median |
| Troponin I | 2 | 0 | 2 | 0.68 | Within‑group median |
| Myoglobin | 1 | 0 | 1 | 0.34 | Within‑group median |
| Hemoglobin | 1 | 1 | 2 | 0.68 | Within‑group median |
| CRP | 1 | 1 | 2 | 0.68 | Within‑group median |
| Uric acid | 2 | 1 | 3 | 1.03 | Within‑group median |
| Cholesterol | 3 | 0 | 3 | 1.03 | Within‑group median |
| Triglycerides | 3 | 0 | 3 | 1.03 | Within‑group median |
| LDL-C | 3 | 0 | 3 | 1.03 | Within‑group median |

CRP: C-reactive protein. LDL-C: Low-density lipoprotein cholesterol.
